# Previous renal support is a predictor for chronic renal replacement therapy after orthotopic liver transplantation

**DOI:** 10.1186/cc10180

**Published:** 2011-06-22

**Authors:** MCC Andreoli, MPV Coelho, ACC Matos, ÉB Rangel, NKG Souza, MÂ Góes, AL Ammirati, TN Matsui, IJ Iizuca, FD Carneiro, ACMS Ramos, MA Souza, RC Afonso, B Ferraz-Neto, MS Durão, MC Batista, JCM Monte, VG Pereira, OFP Santos, BC Santos

**Affiliations:** 1 Einstein Dialysis Center, Albert Einstein Jewish Hospital, São Paulo - SP, Brazil

## Background

In the Model for End-Stage Liver Disease (MELD) era of organ allocation, renal replacement therapy (RRT) has been done in many liver transplant patients. In this setting the time and probability of kidney function recovery is essential for patient and transplant program management.

## Methods

In this study we evaluated a sample of stable post-intensive care dialysis patients from a group of 297 adults who were submitted to orthotopic liver transplantation (OLT) in an urban tertiary medical center from 1 June 2005 to 31 December 2009. We evaluated the average time of renal function recovery (out of need for RRT) in OLT patients on post-intensive care hemodialysis (HD) and determined risk factors for chronic dialysis support during a 1-year follow-up period. Patients were censored at recovery of kidney function, death on HD or end of the follow-up period. The Cox proportional hazards model was used to compare the relative risk (RR) of remaining or not in HD after 1 year and predictor variables.

## Results

We evaluated the clinical records of 83 patients (50 ± 14 years, 64% male, 22% pre-OLT diabetes mellitus (DM), 31% HCV-related disease, MELD 27.5 ± 11.8, 17% acute re-OLT, 37% pre-OLT RRT, pre-OLT serum creatinine 1.5 ± 1.4 mg/dl, 28% pre-OLT proteinuria). During the study period, 70 (84%) patients were removed from dialysis; of these, six (7%) remained on HD for more than 90 days until renal function recovery, 184 days being the longest period required. Nine (11%) patients died on HD and only four (5%) patients were on HD after 1 year. The median of recovery time was 28 days (from 6 to 184 days). Classic risk factors for renal disease, like age and DM, acute re-OLT requirement and pre-OLT RRT, were significant predictors of chronic RRT. In the multivariate analysis, the most important prognostic factor for chronic RRT was the presence of pre-OLT RRT (HR = 1.89, 95% CI = 1.145 to 3.129, *P *= 0.013).

## Conclusion

Given the shortage of available organs, kidney transplantation after or concomitant to OLT must be considered cautiously, especially in OLT patients who were not submitted to pre-OLT RRT.

